# HIV and SARS-CoV-2 Coinfections in Brazil in 2020: Epidemiological, Sociodemographic, and Clinical Characteristics of 36,746 Cases

**DOI:** 10.1590/0037-8682-0126-2024

**Published:** 2024-11-08

**Authors:** Flavia Kelli Alvarenga Pinto, Ronaldo de Almeida Coelho, Elizabeth Moreira Klein, Gerson Fernando Mendes Pereira, Beatriz Gilda Jegerhorn Grinsztejn, Marcos Amaku

**Affiliations:** 1 Universidade de São Paulo, Faculdade de Medicina Veterinária e Zootecnia, Programa de Pós-Graduação em Epidemiologia e Saúde Única, São Paulo, SP, Brasil.; 2 Ministério da Saúde, Departamento de HIV, AIDS, Tuberculose, Hepatites Virais e Infecções Sexualmente Transmissíveis, Brasília, DF, Brasil.; 3 Ministério da Saúde, Departamento de Apoio à Gestão da Atenção Primária, Brasília, DF, Brasil.; 4 Fundação Oswaldo Cruz, Instituto Nacional de Infectologia Evandro Chagas, Rio de Janeiro, RJ, Brasil.

**Keywords:** COVID-19, HIV, Risk factors, Brazil

## Abstract

**Background:**

This study aimed to identify COVID-19 cases among people living with HIV (PLWH) in Brazil in 2020, describe their clinical, sociodemographic, and epidemiological profiles, and evaluate the factors associated with disease severity.

**Methods:**

This cross-sectional study used secondary data obtained from the Brazilian healthcare system. Probabilistic and deterministic data linkage methods were used to identify coinfected patients. Descriptive statistical analysis was conducted, and factors associated with severe cases were evaluated using Pearson's chi-squared test, Student's t-test, and logistic regression.

**Results:**

In 2020, 36,746 coinfections were identified, making it one of the largest coinfection databases described worldwide. In total, 4,502 (12.25%) patients had severe cases and 32,244 (87.75%) had non-severe cases. The covariates age (OR=1.05; 95% CI: 1.05-1.06), nonwhite ethnicity (OR=1.68; 95% CI: 1.56-1.81), history of AIDS diagnosis (OR=1.17; 95% CI: 1.08-1.28), recent HIV diagnosis (OR=5.47; 95% CI: 4.25-7.02), absence of antiretroviral therapy (OR=1.70; 95% CI: 1.57-1.84), CD4+ < 200 (OR=6.41; 95% CI: 5.09-8.08), detectable HIV viral load (OR=2.61; 95% CI: 2.21-3.05), ≥ 1 comorbidity (OR=4.09; 95% CI: 3.79-4.41), and ≥ 4 symptoms were associated with increased severity.

**Conclusions:**

Multiple factors were linked to severe COVID-19, including uncontrolled HIV infection, age > 50 years, comorbidities, and racial disparities. This study reinforces the importance of maintaining public policies focused on early HIV diagnosis, access and adherence to treatment, especially for minority ethnic groups, and focusing on premature aging in PLWH.

## INTRODUCTION

In early 2020, several countries faced the spread of the coronavirus disease 2019 (COVID-19) caused by severe acute respiratory syndrome coronavirus 2 (SARS-CoV-2), which was first identified in Wuhan, China^1^. This rapid spread prompted the World Health Organization (WHO) to declare a pandemic in March 2020^2^.

In Brazil, the response to the pandemic was initially based on classical epidemiological control and prevention measures, including quarantine, isolation, hygiene practices, monitoring, and surveillance. To monitor variations in the incidence of COVID-19, the country used mandatory case registration in two health information systems^3^. Asymptomatic, mild, and moderate (non-severe) cases were recorded using the e-SUS Notifica information system. Whereas severe and critical cases requiring hospitalization, as well as deaths regardless of hospitalization, were recorded using the Influenza Epidemiological Surveillance Information System (SIVEP-GRIPE)^3^.

Analysis of these records allows tracking of disease incidence and understanding the demographic, social, and epidemiological profiles of patients^4^. Monitoring cases in Brazil and similar data from other countries show that severe COVID-19 cases are more frequent among those with comorbidities, such as heart disease, diabetes mellitus, or respiratory disease^4^ ^,^ ^5^. Other risk factors such as age ≥ 60 years, obesity, and immunosuppression, including people living with HIV (PLWH), are also significant^3^.

Although some studies have shown clinical similarities between PLWH and the general population with respect to COVID-19^6^ ^,^ ^7^, immunosuppression is concerning because it renders individuals more vulnerable to a multitude of infections^8^. Infection with human immunodeficiency virus (HIV) compromises the immune system, leading to a deficiency in antibody production or T-cell function and increasing susceptibility to opportunistic infections that are often fatal and difficult to treat^8^. Studies on COVID-19 have shown that CD4+ T cells are vital for immune responses, activating B cells for antibodies, and aiding CD8+ T cells to eliminate SARS-CoV-2. Patients with severe COVID-19 exhibit reduced CD4+ T cell counts, leading to prolonged viral replication and a higher susceptibility to secondary infections^9^.

In this context, reducing severe cases and deaths from COVID-19 also involves providing care to PLWH, especially those with low CD4+ T cell counts, high HIV viral loads, and not undergoing antiretroviral therapy (ART). In 2021, an estimated 960,000 PLWH were recorded in Brazil, among whom approximately 108,000 had not been diagnosed. Among those diagnosed, 256,000 were aged > 50 years^10^, which may imply more chronic conditions^11^ and a greater risk of severe COVID-19^1^.

Moreover, Brazil, among other countries, has adhered to the United Nations Millennium Development Goals, which include combating HIV/AIDS. The goals include diagnosing 90% of all HIV-positive individuals, providing ART to 90% of those diagnosed, and achieving viral suppression in 90% of those treated^12^. Well-defined care and management policies are required to improve the quality of life of PLWH and prevent the spread of HIV.

Considering the National Sexually Transmitted Diseases and AIDS Control Program and the stage of knowledge regarding COVID-19 among PLWH, understanding their outcomes to support appropriate health policies for this population is essential in Brazil and worldwide. Therefore, this study aimed to identify HIV/SARS-CoV-2 coinfected patients registered in Brazilian health information systems in 2020 and describe their clinical, sociodemographic, and epidemiological characteristics, with a focus on identifying factors associated with the most severe cases.

## METHODS

###  • Research Design and Population 

This was a cross-sectional study utilizing secondary databases from Brazilian health information systems. The study population comprised HIV/SARS-CoV-2 coinfected patients in Brazil identified through database linkage who were diagnosed with COVID-19 in 2020.

###  •Identification of HIV/SARS-CoV-2 Coinfection 

**Data Linkage:** For database linkage, cases of PLWH were selected based on the Ministry of Health's criteria for diagnosing HIV within clinical disease monitoring^10^. In the SIVEP-GRIPE and e-SUS Notifica system databases, the first SARS-CoV-2 infection in each patient with positive laboratory or clinical-epidemiological evidence was selected. Furthermore, for probabilistic linkage, only cases that had personal identification information (PI), patient’s name, mother’s name, and date of birth were selected. For the deterministic method, the complete individual taxpayer registry number (CPF) accompanied by at least one PI was needed.

First, a deterministic linkage was performed between the HIV/AIDS and e-SUS Notifica system databases (non-severe cases of COVID-19), followed by the HIV/AIDS and SIVEP-GRIPE databases (severe cases of COVID-19). Cases with identical CPFs and at least one identical PI field were considered as valid.

Next, probabilistic linkage was conducted using an automated duplicate analysis process in RecLink III software. For this purpose, two databases were constructed: HIV/AIDS with the e-SUS Notifica system (non-severe COVID-19) and HIV/AIDS with SIVEP-GRIPE (severe COVID-19), containing PI and variable standardization for the Soundex codes of the first and last names of the cases. To verify accuracy, the following parameters were used: patient's name (98% accuracy, 0.0011% error, and 85% threshold), mother's name (74% accuracy, 0.0046% error, and 85% threshold), and date of birth (98% accuracy, 2.356% error, and 65% threshold); these parameters are similar to those used by the Brazilian Ministry of Health for HIV/AIDS database construction^17^. Finally, homonyms with more than four pairs were excluded, and pairs with at least one identical PI field were selected.

###  •Analysis of HIV/SARS-CoV-2 Coinfection: 

All patients diagnosed with COVID-19 after 2020 were excluded from the analysis, as were those diagnosed with HIV more than 30 days after COVID-19, and those diagnosed with AIDS more than 5 months after COVID-19. The criteria for the last two cases were based on the natural histories of HIV and AIDS^12^.

In cases where two COVID-19 infections occurred (severe and non-severe COVID-19) within an interval ≥ 90 days, only the first was considered^18^. Those with an interval of less than 90 days were considered to have the same infection^18^, and their symptoms and comorbidities were confirmed.

The variables of date of birth, sex, education, and ethnicity were obtained from the reported HIV/AIDS cases^14^. Cases without this information were supplemented with data from other databases^15^ ^,^ ^16^. Demographic variables used information from COVID-19 cases^15^ ^,^ ^16^, and in the absence of information, data from the HIV/AIDS cases^14^ were considered. As the e-SUS Notifica database for patients with non-severe COVID-19 had only affirmative responses (yes)^15^, dichotomous variables from all databases without information were deemed negative (no).

Considering the criteria adopted by the Brazilian government, in which severe and critical hospitalized cases of COVID-19 are notified in the SIVEP-GRIPE with the SARS database and asymptomatic, mild, and moderate cases are registered in the e-SUS Notifica database^3^, we designated the notification variable type ‘SARS’ as “severe cases of COVID-19” and the others, notified in the e-SUS Notifica database, as “non-severe cases of COVID-19.” Therefore, we used severe cases of COVID-19 as the independent variable to identify the factors associated with the severity of HIV/SARS-CoV-2 coinfection.

Univariate descriptive statistical analyses were performed to verify the distribution of the variables. The mean and standard deviation were calculated for the ‘age’ variable. Furthermore, the possible associations between the studied variables and outcomes (severe and critical hospitalized patients) were assessed using Pearson's chi-squared test, and Student's t-test was applied for the ‘age’ variable outcome. Furthermore, Cramer’s*V test was performed to calculate the* effect sizes for the chi-squared test. Magnitudes of 0.1, 0.3, and 0.5 were considered small, medium, and large, respectively^19^.

Given that the outcome of interest was uncommon in the study population, the adjusted odds ratio (OR) derived from logistic regression was close to the risk ratio or prevalence ratio^20^. A multivariate logistic regression model was used that included independent variables associated with the outcome from univariate analysis, such as age, nonwhite ethnicity, years of schooling, history of AIDS diagnosis, recent HIV diagnosis, ART, detectable HIV viral load, one or more comorbidities (unrelated to HIV/AIDS), and four or more COVID-19 symptoms, at a p value ≤ 0.20. Variables with a significance level of ≤ 5% were retained in the final adjusted model, and for each predictor variable adjusted for the others, the OR with a 95% confidence interval (CI) was estimated. All model adequacy tests were conducted.

The following variables were removed from the univariate and multivariate statistical analysis: final evolution, HIV exposure category, and immunosuppression as a comorbidity, due to the lack of information in some of their categories or because they could be explained by other variables already included in the model according to the collinearity criterion. In addition, the ‘region of residence’ variable was excluded because its detailed analysis requires a spatial study, which should be based on another modeling strategy.

Deterministic linkages and other analyses were conducted using SPSS Statistics 18 and R 4.2.3 software. The database linkage process was part of the activities performed by researchers within the Department of HIV/AIDS, Tuberculosis, Viral Hepatitis, and Sexually Transmitted Infections of the Brazilian Ministry of Health, and adhered to the ethical principles of confidentiality inherent to their functional responsibilities. This study was approved by the Ethics Committee of the Clinics Hospital of the University of São Paulo Medical School (No. 6,741,713) through Plataforma Brazil.

## RESULTS

**FIGURE 1:**
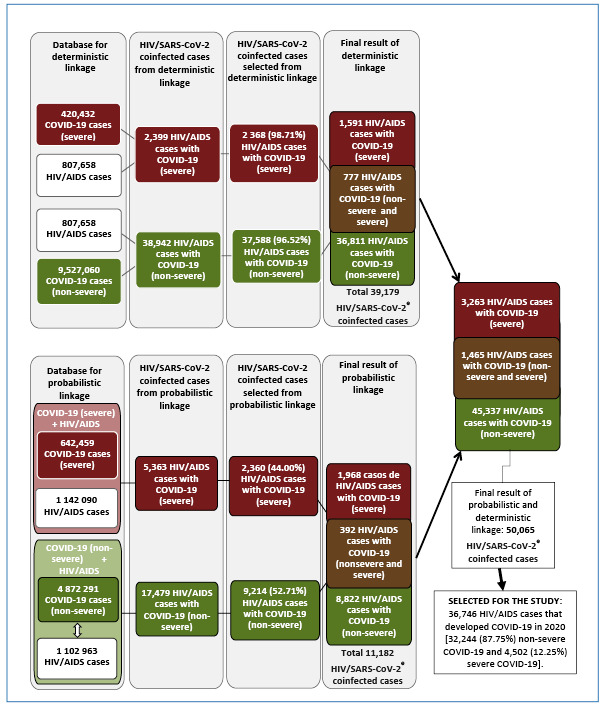
Diagram illustrating the data linkage process between the HIV/AIDS^a^ and the COVID-19^b^ databases (non-severe^c^ and severe^d^ cases). Brazil, 2020.

^a^ **HIV/AIDS**: human immunodeficiency virus and/or acquired immunodeficiency syndrome. ^b^ **COVID-19**: Coronavirus Disease 2019. ^c^ **Non-severe**: asymptomatic, mild, and moderate COVID-19 cases registered in the e-SUS Notifica database. ^d^ **Severe:** severe and critically hospitalized COVID-19 cases as well as deaths regardless of hospitalization registered in the SIVEP-GRIPE database. ^e^ **HIV/SARS-CoV-2**: coinfection with HIV and SARS-CoV-2. **Source:** Prepared by the authors from the study results.

A deterministic linkage was performed between 807,658 HIV/AIDS cases and 420,432 patients with severe COVID-19, resulting in 2,399 HIV/AIDS cases with severe COVID-19. Of these, 2,368 (98.71%) were selected for the study because of their CPF number and at least one identical PI variable. Another deterministic linkage involved 807,658 HIV/AIDS cases and 9,527,060 asymptomatic, mild, or moderate COVID-19 cases (or non-severe COVID-19 cases), resulting in 38,942 cases. Of these, 37,588 (96.52%) were included in this study. In total, 39,179 cases of HIV/AIDS with COVID-19 were identified, with 777 (1.98%) doubly reported as non-severe and severe cases of COVID-19 (Figure 1).

The probabilistic linkage between 1,142,090 HIV/AIDS and 642,459 severe COVID-19 cases resulted in 5,363 HIV/AIDS cases with severe COVID-19. Of these, 2,360 (44.00%) were selected after validation. The subsequent probabilistic linkage involved 1,102,963 HIV/AIDS cases and 4,872,291 asymptomatic, mild, or moderate COVID-19 cases (or non-severe COVID-19 cases), resulting in 17,479 HIV/AIDS cases with asymptomatic, mild, or moderate COVID-19. Of these, 9,214 (52.71%) were included in the study. Overall, 11,182 cases of HIV/AIDS with COVID-19 were identified, 392 (3.5%) of which were reported as non-severe or severe cases of COVID-19 (Figure 1).

The combination of these related databases identified 296 additional cases doubly identified as non-severe and severe cases of COVID-19, resulting in 50,065 HIV and SARS-CoV-2 coinfection cases. Among these, 1,465 were reported as non-severe and severe cases of COVID-19, 3,263 as severe COVID-19 cases (severe and critical, hospitalized COVID-19 cases), and 45,337 as non-severe COVID-19 cases (asymptomatic, mild, or moderate COVID-19 cases) (Figure 1).

In this study, 50,065 individuals coinfected with HIV/SARS-CoV-2 were identified, of whom 36,746 were selected because they developed COVID-19 in 2020. Among the 2020 cases, 32,244 (87.75%) had non-severe COVID-19, whereas 4,502 (12.25%) progressed to severe COVID-19. Of the 36,746 coinfected individuals, only 4,149 (11.29%) remained asymptomatic.

**FIGURE 2:**
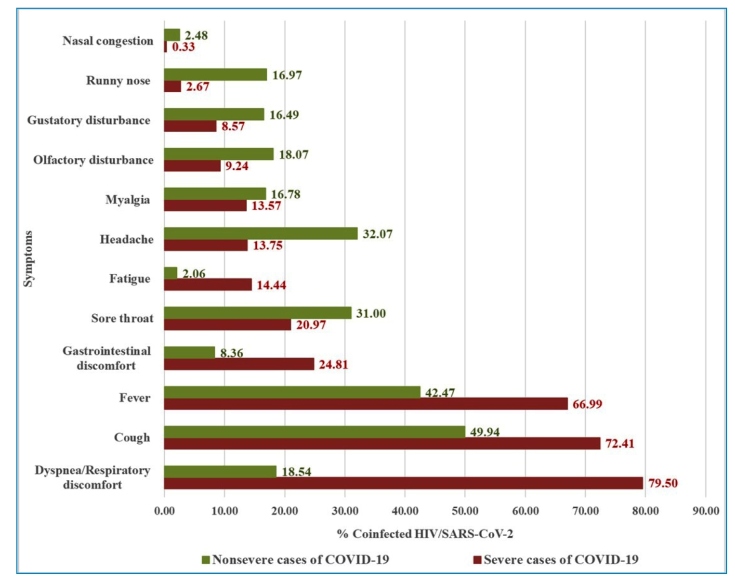
Proportion of individuals coinfected with HIV/SARS-CoV-2^a^ (non-severe^b^ and severe^c^ cases of COVID-19^d^) stratified by reported symptoms. Brazil, 2020.

^a^ HIV/SARS-CoV-2: coinfection with human immunodeficiency virus (HIV) and severe acute respiratory syndrome coronavirus 2 (SARS-CoV-2). ^b^ Non-severe: asymptomatic, mild, and moderate COVID-19 cases. ^c^ Severe: severe and critically hospitalized COVID-19 cases as well as deaths, regardless of hospitalization. ^d^ COVID-19: Coronavirus Disease 2019. **Source:** Prepared by the authors from the study results.

Among the symptoms evaluated, the four most frequent in severe cases were dyspnea (79.50% of cases), followed by cough (72.41%), fever (66.99%), and gastrointestinal disorders (24.81%). In mild and moderate cases, the most prevalent complaints were cough (49.94%), fever (42.47%), headache (32.07%), and sore throat (31.00%) (Figure 2).

**FIGURE 3:**
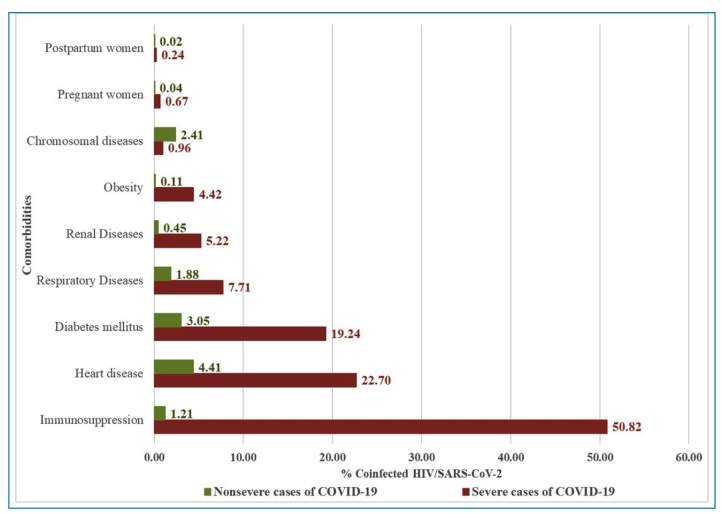
Proportion of individuals coinfected with HIV/SARS-CoV-2^a^ (non-severe^b^ and severe^c^ cases of COVID-19^d^) stratified by reported comorbidities. Brazil, 2020.

^a^ HIV/SARS-CoV-2: coinfection with human immunodeficiency virus (HIV) and severe acute respiratory syndrome coronavirus 2 (SARS-CoV-2). ^b^ Non-severe: asymptomatic, mild, and moderate COVID-19 cases. ^c^ Severe: severe and critically hospitalized COVID-19 cases as well as deaths, regardless of hospitalization. ^d^ COVID-19: Coronavirus Disease 2019. **Source:** Prepared by the authors from the study results.

Regarding comorbidities, 30,206 (82.20%) patients did not report any of the evaluated comorbidities. Among the 30,206 cases, 95.62% were non-severe COVID-19 cases. Immunosuppression was the most common comorbidity among patients with severe COVID-19, and was present in 2,288 (50.82%) patients, followed by heart disease (22.70%), diabetes mellitus (19.24%), and respiratory diseases (7.71%). In non-severe cases, the most frequent comorbidities were heart disease (4.42%), diabetes mellitus (3.05%), chromosomal diseases (2.41%), and respiratory diseases (1.88%) (Figure 3).

As shown in Table 1, the average age among coinfected patients was 42.3 years, with a standard deviation (SD) of 12.4 years, which was greater (49.7 years and SD=13.4) among patients with severe COVID-19. Most patients were men, totaling 25,479 (69.34%). Of those affected, 15,011 (40.85%) resided in southeastern Brazil. The most frequent category of HIV exposure was sexual exposure, with 12,365 (33.65%) participants being heterosexual. Non-white ethnicity was the most common ethnicity, representing 20,408 (55.54%) patients. Among the non-severe COVID-19 patients, 14,846 (46.04%) were white individuals, while among the severe cases, this percentage decreased to 33.14%. Regarding education, 10,854 (29.54%) patients had ≥ 9 years of schooling; however, this number decreased to 17.82% in the severe case group. In total, 27,851 (75.79%) patients had a history of AIDS, which increased to 78.52% among severe cases. A recent HIV diagnosis was verified in 361 (0.98%) cases, with a higher percentage (3.95%) among severe cases. Furthermore, 8,062 (21.94%) patients did not undergo ART, and this percentage was even greater (33.56%) in critically ill patients. A total of 448 (1.22%) patients had a CD4+ T-cell count of < 200 cells/mm^3^, with a greater percentage (5.94%) in the severe group. A detectable HIV viral load was observed in 1,196 patients (3.25%); however, this percentage reduced to 2.58% among non-severe cases and increased to 8.11% in severe cases. A total of 5,160 (14.04%) patients reported having one or more comorbidities, and this percentage was nearly three times (40.52 %) among patients with severe COVID-19. Notably, 13,983 (38.05%) patients reported four or more symptoms, reaching 56.95% in the severe group. Regarding the evolution of cases, the majority, 19,265 (52.43%) were cured, while 1,414 (3.85%) died, with 1,352 (30.03%) severe cases dying.

**TABLE 1:** Epidemiological, sociodemographic, and clinical characteristics of individuals coinfected with HIV/SARS-CoV-2^a^ stratified by COVID-19 severity. Brazil, 2020.

Variables		Coinfected HIV/SARS-CoV-2	COVID-19 cases		P value	Cramér's V
			Non-severe^b^	Severe^c^		
		N=36,746	N=32,244	N=4,502		
**Age**						
Mean (Standard Deviation)		42.3 (SD=12.4)	41.3 (SD=11.9)	49.7 (SD=13.4)	<0.001	-
**Sex**					0.78	0.001
Women		11,267 (30.66%)	9,878 (30.64%)	1,389 (30.85%)		
Men		25,479 (69.34%)	22,366 (69.36%)	3,113 (69.15%)		
**Residence Region**					<0.001	0.053
Center West		3,178 (8.65%)	2,822 (8.75%)	356 (7.91%)		
Northeast		7,461 (20.30%)	6,459 (20.03%)	1,002 (22.26%)		
North		4,009 (10.91%)	3,576 (11.09%)	433 (9.62%)		
South		7,087 (19.29%)	6,422 (19.92%)	665 (14.77%)		
Southeast		15,011 (40.85%)	12,965 (40.21%)	2,046 (45.44%)		
**Category of HIV exposure**						
Sexual	Bisexual	1,940 (5.28%)	1,703 (5.28%)	237 (5.27%)	<0.001	0.089
	Heterosexual	12,365 (33.65%)	10,614 (32.92%)	1,751 (38.89%)		
	Homosexual	8,334 (22.68%)	7,724 (23.95%)	610 (13.55%)		
Blood	Injectable drug	701 (1.90%)	560 (1.74%)	141 (3.13%)		
	Transfusion/Hemophiliac	32 (0.09%)	21 (0.07%)	11 (0.25%)		
	Biological Accident Material	7 (0.02%)	7 (0.02%)	0 (0.00%)		
	Vertical Transmission	274 (0.75%)	245 (0.76%)	29 (0.64%)		
	No information	13,093 (35.63%)	11,370 (35.26%)	1,723 (38.27%)		
**Ethnicity**					<0.001	0.085
White		16,338 (44.46%)	14 846 (46.04%)	1,492 (33.14%)		
Nonwhite		20,408 (55.54%)	17 398 (53.96%)	3,010 (66.86%)		
**Schooling (years of study)**					<0.001	0.117
< 5 years		2,453 (6.68%)	1,918 (5.95%)	535 (11.88%)		
5 to 8 years		7,542 (20.52%)	6,425 (19.93%)	1,117 (24.81%)		
9 years or more		10,854 (29.54%)	10,052 (31.17%)	802 (17.82%)		
No information		15,897 (43.26%)	13,849 (42.95%)	2,048 (45.49%)		
**History of AIDS diagnosis** ^f^					<0.001	0.024
No		8,895 (24.21%)	7,928 (24.59%)	967 (21.48%)		
Yes		27,851 (75.79%)	24,316 (75.41%)	3,535 (78.52%)		
**Recent HIV diagnosis** ^g^					<0.001	0.112
No		36,385 (99.02%)	32,061 (99.43%)	4,324 (96.05%)		
Yes		361 (0.98%)	183 (0.57%)	178 (3.95%)		
**In use of ART** ^h^					<0.001	0.105
Yes		28,684 (78.06%)	25,693 (79.68%)	2,991 (66.44%)		
No		8,062 (21.94%)	6,551 (20.32%)	1,511 (33.56%)		
**CD4 < 200 cells/mm3** ^i^					<0.001	0.160
No		36,298 (98,78%)	32,063 (99.44%)	4,235 (94.06%)		
Yes		448 (1.22%)	181 (0.56%)	267 (5.94%)		
**Detectable HIV viral load** ^j^					<0.001	0.102
Yes		35,550 (96.75%)	31,413 (97.42%)	4,137 (91.90%)		
No		1,196 (3.25%)	831 (2.58%)	365 (8.10%)		
**Qty Comorbidities (except immunosuppressed)**					<0.001	0.284
none		31,586 (85.96%)	28,908 (89.65%)	2,678 (59.48%)		
1 or more		5,160 (14.04%)	3,336 (10.35%)	1,824 (40.52%)		
**Qty Symptoms**					<0.001	0.145
< 4		22,763 (61.95%)	20,825 (64.59%)	1,938 (43.05%)		
4 or more		13,983 (38.05%)	11,419 (35.41%)	2,564 (56.95%)		
**Evolution of coinfection**					<0.001	0.539
Cure		19,265 (52.43%)	16,566 (51.38%)	2,699 (59.95%)		
Death		1,414 (3.85%)	62 (0.19%)	1,352 (30.03%)		
Death from other causes		18 (0.05%)	0 (0.00%)	18 (0.40%)		
No information		16,049 (43.67%)	15,616 (48.43%)	433 (9.62%)		

^a^ HIV/SARS-CoV-2: coinfection with human immunodeficiency virus (HIV) and severe acute respiratory syndrome coronavirus 2 (SARS-CoV-2). ^b^ Non-severe: asymptomatic, mild, and moderate COVID-19 cases. ^c^ Severe: severe and critically hospitalized COVID-19 cases as well as deaths, regardless of hospitalization. ^d^ P value of Pearson's chi-squared test for categorical variables and Student's t-test for age. ^e^ Effect sizes for the chi-squared test: 0.1 small, 0.3 medium, and 0.5 large. ^f^ PLWH with a history of at least one CD4 result of ≤ 350 cells/mm^3^ or having been classified with AIDS by the CDC/Rio de Janeiro/Caracas criteria. ^g^ Person diagnosed with HIV 30 days before or after COVID-19 diagnosis or AIDS 30 days before or 150 days after COVID-19 diagnosis. ^h^ PLWH who received at least one ART at an interval of ≤ 90 days before contracting COVID-19. ^i^ PLWH with at least one CD4+ T cell count of < 200 cells/mm^3^ within 30 days before or after contracting COVID-19. ^j^ PLWH with at least one detectable CV of > 500 copies/mL within 90 days before or after COVID-19 diagnosis. **Source:** Prepared by the authors from the study results.

The logistic regression model estimating the factors associated with the risk of severe and critical hospitalized cases of COVID-19 among HIV/SARS-CoV-2 coinfected individuals, along with their respective raw and adjusted OR, is presented in Table 2. Among the factors associated with the severity of COVID-19 according to the simple regression analysis, older age was associated with a greater risk. Whereas, ≥ 5 years of schooling was a protective factor. Nonwhite ethnicity, history of AIDS, recent HIV diagnosis, absence of ART, CD4+ T cell counts of < 200 cells/mm^3^, detectable HIV viral load, having at least one comorbidity (not related to HIV/AIDS), and ≥ 4 COVID-19 symptoms increased the risk of a severe case.

**TABLE 2:** Risk factors associated with severe COVID-19 in individuals coinfected with HIV/SARS-CoV-2^a^. Brazil, 2020.

Variables	OR Crude [95% CI^b^]	p value^c^	OR Adjusted [95% CI^b^]	p value^d^
**Age** * *				
Age (continuous variable)	1.05 [1.05, 1.06]	<0.0001	1.05 [1.05, 1.06]	<0.0001
**Race**				
White	Ref		Ref	
Nonwhite	1.72 [1.61, 1.84]	<0.0001	1.68 [1.56, 1.81]	<0.0001
**Schooling (years of study)**				
< 5 years	Ref		Ref	
5 to 8 years	0.62 [0.56, 0.70]	<0.0001	0.88 [0.78, 1.01]	0.085
9 years or more	0,29 [0.25, 0.32]	<0.0001	0.67 [0.58, 0.76]	<0.0001
No information	0.53 [0.48, 0.59]	<0.0001	0.90 [0.79, 1.02]	0.074
**History of AIDS diagnosis** ^e^				
No	Ref		Ref	
Yes	1.19 [1.11, 1.29]	<0.0001	1.17 [1.08, 1.28]	<0.0001
**Recent diagnosis HIV** ^f^				
No				
Yes	7.21 [5.85, 8.89]	<0.0001	5.47 [4.25, 7.02]	<0.0001
**In use of ART** ^g^				
Yes	Ref		Ref	
No	1.98 [1.85, 2.12]	<0.0001	1,70 [1.57, 1.84]	<0.0001
**CD4 < 200 cells/mm3** ^h^				
No	Ref		Ref	
Yes	11.17 [9.22, 13.52]	<0.0001	6.41 [5.09, 8.08]	<0.0001
**Detectable HIV viral load** ^i^				
No	Ref		Ref	
Yes	3.34 [2.93, 3.79]	<0.0001	2.61 [2.21, 3.05]	<0.0001
**Qty Comorbidities (except immunosuppressed)**				
None	Ref		Ref	
1 or more Comorbidities	5.90 [5.51, 6.33]	<0.0001	4.09 [3.79, 4.41]	<0.0001
**Qty Symptoms**				
< 4	Ref		Ref	
4 or more	2.41 [2.26, 2.57]	<0.0001	2.52 [2.48, 2.70]	<0.0001

^a^ HIV/SARS-CoV-2: coinfection with human immunodeficiency virus (HIV) and severe acute respiratory syndrome coronavirus 2 (SARS-CoV-2). ^b^ 95% confidence interval. ^c^ p value according to the univariate logistic regression model. ^d^ p value according to the multivariate logistic regression model. ^e^ PLWH with a history of at least one CD4 result ≤ 350 cells/mm^3^ or having been classified with AIDS by the CDC/Rio de Janeiro/Caracas criteria. ^f^ Persons diagnosed with HIV 30 days before or after COVID-19 or AIDS 30 days before or 150 days after COVID-19. ^g^ PLWH who received at least one ART at an interval of ≤ 90 days before contracting COVID-19. ^h^ PLWH with at least one CD4+ T cell count of < 200 cells/mm^3^ within 30 days before or after contracting COVID-19. ^i^ PLWH with at least one detectable CV of > 500 copies/mL within 90 days before or after COVID-19. **Source:** Prepared by the authors from the study results.

According to multiple analyses, ≥ 9 years of schooling remained a protective factor. Covariates age (OR=1.05; 95% CI: 1.05, 1.06), nonwhite ethnicity (OR=1.68; 95% CI: 1.56, 1.81), history of AIDS diagnosis (OR=1.17; 95% CI: 1.08, 1.28), recent HIV diagnosis (OR=5.47; 95% CI: 4.25, 7.02), absence of ART (OR=1.70; 95% CI: 1.57, 1.84), CD4+ T cell counts of < 200 cells/mm^3^ (OR=6.41; 95% CI: 5.09, 8.08), detectable HIV viral load (OR=2.61; 95% CI: 2.21, 3.05), ≥ 1 comorbidity (OR=4.09; 95% CI: 3.79, 4.41), and ≥ 4 COVID-19 symptoms (OR=2.52; 95% CI: 2.48, 2.70) were associated with an increased risk of severe COVID-19, as determined by simple regression analysis. However, 5-8 years of schooling or no information on education level were not associated with severity in coinfected individuals after adjusting the multiple regression model (Table 2).

## DISCUSSION

In this study, we used secondary data from an extensive cohort of more than one million PLWH and employed linkage methods to identify those who contracted COVID-19 in Brazil in 2020. We identified a significant number of HIV/SARS-CoV-2 coinfections, with a prevalence of 12.25% severe cases. We observed a combination of factors associated with disease severity, particularly those related to uncontrolled HIV infection and racial disparities. This study contributes to the knowledge of HIV/SARS-CoV-2 coinfection and underscores the importance of maintaining public policies focused on HIV control and prioritizing minority ethnic groups.

The number of cases of coinfected individuals that we identified was one of the highest recorded compared to other studies^6^ ^,^ ^21^ ^-^ ^24^. Data linkage is a feasible, low-cost strategy capable of improving information quality^25^. Although most coinfection cases were identified deterministically, the use of probabilistic methods has also played a significant role in increasing case detection. Similarly, the 2020 epidemiological bulletin indicated that this method was used by 30.8% of PLWH in the country^17^.

The most common symptoms observed in the severe cases were dyspnea, cough, and fever. These clinical manifestations of COVID-19 in PLWH resemble those observed in the general population^7^. Cardiac disease and diabetes mellitus (comorbidities not associated with HIV) were the most prevalent. Additionally, we found that those with ≥ 4 symptoms and at least one comorbidity were at greater risk of severe disease. These comorbidities, previously known as risk factors for COVID-19^5^, especially cardiopulmonary diseases and diabetes mellitus, have been described as severity factors in PLWH^7^ ^,^ ^26^.

Advanced age, which has been associated with complications and death since the early reports on COVID-19^1^, was also found to be a risk factor for severe disease among PLWH in this study. Although Brazil considers individuals aged > 60 years to be at high risk for COVID-19 complications^3^, we found that the average age of severely coinfected patients was 49.7 years. Notably, despite advances in ART that have made HIV/AIDS a manageable chronic disease, PLWH continue to face metabolic and immune system issues, contributing to premature aging and increased disease and death risk^27^.

Non-white PLWH were more susceptible to severe forms of COVID-19, whereas those with higher levels of education had a lower risk of developing severe COVID-19. These results are consistent with other studies that have indicated that PLWH with lower education levels, as well as those identified as mixed ethnicity, Hispanic, and especially black individuals, have a greater likelihood of progressing to severe COVID-19^21^ ^,^ ^28^. These disparities reflect the broader issues of socioeconomic inequality, lifestyle factors, and pre-existing health conditions that influence disease severity^29^.

Individuals recently diagnosed with HIV or those at a more advanced stage and had a history of AIDS were also identified as having a greater chance of progression to severe COVID-19. This study did not determine the stage of HIV infection in individuals who were recently diagnosed, but it is important to highlight that individuals may develop acute retroviral syndrome (ARS), which is characterized by viremia and various clinical manifestations, up to four weeks after HIV virus exposure ^12^ ^,^ ^30^. During the pandemic, regardless of ARS symptoms, individuals often experienced delayed HIV diagnosis owing to healthcare services and laboratories focusing primarily on SARS-CoV-2^31^. However, after this phase, HIV can become asymptomatic for years, potentially delaying diagnosis and further compromising the immune system, resulting in increased susceptibility to opportunistic infections^8^ ^,^ ^12^ ^,^ ^32^.

Furthermore, we observed that the absence of ART, an unsuppressed HIV viral load, and CD4+ T cell counts of < 200 cells/mm^3^ were associated with a significant increase in the risk of progression to severe COVID-19. Other studies have indicated that these factors, which are related to uncontrolled HIV infection, are widely associated with complications and deaths from COVID-19^21^ ^-^ ^23^ ^,^ ^26^. Notably, despite ART not showing beneficial effects among individuals infected with SARS-CoV-2 and clinical trial results remaining conflicting and scarce^9^ ^,^ ^22^, its use can lead to an undetectable HIV viral load, ensuring nearly complete immune recovery in PLWH^22^.

Combination of various factors increases the risk of severe COVID-19, especially those associated with immunodeficiency or uncontrolled HIV infection, as well as comorbidities and racial differences^21^ ^,^ ^22^. However, many of these findings are from a limited number of studies, including controlled studies, owing to small sample sizes and a focus on hospitalized cases. In contrast, our study, which described more than 36,000 cases, the majority of whom were not hospitalized, corroborated these findings by demonstrating that multiple factors were associated with severity risk. Moreover, the use of data linkage techniques can yield important results.

This study has limitations owing to the use of fragmented secondary data that may contain gaps such as underreporting, inconsistencies, and incompleteness. The vaccination status was not assessed because COVID-19 vaccines were unavailable in Brazil during the study period. Unfortunately, laboratory information on the HIV viral load and CD4+ T cell count for individuals treated in the private healthcare network, as well as data on AIDS-related opportunistic infections, were not available. The validation criteria for data linkages were highly specific, and potentially underestimated the number of cases. However, this study provides one of the first broader insights into the clinical, sociodemographic, and epidemiological status of PLWH with COVID-19 in Brazil.

In conclusion, multiple severity factors influence COVID-19 in PLWH, and uncontrolled HIV infection stands out. Average age of < 60 years in severe cases highlights accelerated aging among PLWH, reinforcing the need to consider PLWH aged ≥ 50 years in health policies for older individuals. The identified ethnic disparities underscore the importance of monitoring strategies that prioritize minority ethnic groups. These findings support the development of studies with the potential to benefit from the use of data linkage methods across different data sources, and emphasize the importance of public policies that prioritize expanding HIV diagnosis, early access to ART, and treatment adherence.

## Data Availability

The databases used included the HIV/AIDS database compiled for the HIV/AIDS-2020 bulletin^13^, which encompasses cases reported in the Notifiable Diseases Information System (Sinan) and three other health systems^14^; the e-SUS Notifica system database, containing notifications of asymptomatic, mild, and moderate cases of COVID-19^3^ ^,^ ^15^ until April 27, 2021; and the SIVEP-GRIPE database, containing notifications of hospitalized severe and critical cases, as well as deaths from COVID-19^3^ ^,^ ^16^ until February 15, 2021.
